# Asymptomatic polyvascular atherosclerotic disease requires a complex management strategy – case report and review of the literature

**Published:** 2009

**Authors:** R Jurcut, A Florian, L Zarma, I Arsenescu, M Rosca, A Calin, C Ginghina

**Affiliations:** * Department of Cardiology, “Carol Davila” University of Medicine and Pharmacy, “Prof. Dr. C.C.Iliescu” Institute of Cardiovascular Diseases

## Abstract

Atherosclerosis represents a systemic disease that affects all major vascular territories. Despite advances in medical therapies to prevent atherosclerosis and better manage patients with established peripheral arterial disease (PAD), the incidence of PAD continues to increase, and associated morbidity remains high, especially as the population ages. Over the past decade, percutaneous revascularization therapies for the treatment of patients with PAD have tremendously evolved, and a great number of patients can be offered treatment options that are less invasive than traditional surgical ones.

Here we are presenting the case of a 67-year old diabetic woman with multiple cardiovascular risk factors and oligosymptomatic atherosclerotic involvement in several important territories (severe internal carotid stenosis, severe proximal left subclavian artery stenosis, critical serial stenosis in the mid-segment of the left anterior descending artery). Bilateral staged carotid artery plus left subclavian artery stenting was performed with very good results. Regarding the existence of asymptomatic one vessel coronary artery disease (CAD) with a negative exercise test our attitude was to maximize anti-ischemic medical therapy.

In conclusion, the presence of multivascular atherosclerotic disease in a diabetic patient with coexisting risk factors is not surprising and it only reinforces the well known fact that we have to search for the involvement of other territories in an oligosymptomatic patient.

## Introduction

Atherosclerosis is one of the most important and common causes of death and disability throughout the world. During the last half of the past century, coronary artery atherosclerosis has been a major focus for basic and clinical investigation, yet atherosclerosis is a systemic disease with important sequelae in many other regional circulations. Moreover, once the disease is apparent in one vascular territory, there is increased risk for adverse events in other territories. For example, patients with peripheral arterial disease have a 4-fold greater risk of myocardial infarction and a 2- to 3-fold greater risk of stroke than patients without peripheral arterial disease [**1**].

Carotid artery disease is the main cause of ischemic stroke, the risk of which is directly related to the severity of stenosis and presence of symptoms. In Western countries, stroke is the third leading cause of death, after heart disease and cancer, and is the most common cause of permanent disability. This neurological condition affects 0.2% of the population each year, and the incidence of stroke-related death is expected to double over the next 30 years [**2**].

The prevalence of asymptomatic extracranial carotid stenosis (≥ 50%) in persons > 65 years of age is estimated to be between 5% and 10%, whereas only ≤ 1% of the patients are estimated to have a severe narrowing (>80%) [**3**]. In asymptomatic patients with ≥ 50% carotid artery stenoses, the annual risk of stroke is between 1% and 4.3% [**4**]. The asymptomatic patients at highest risk of stroke are those with more severe stenosis and those with progressive carotid artery stenosis [**4**]. With an asymptomatic carotid stenosis of >75%, the natural history risk of having a stroke may be as high as 5.5% per year [**5**].

The advantage of carotid endarterectomy (CEA) over medical therapy in patients with significant carotid stenoses has been established in randomized studies [**6**]. Carotid artery stenting (CAS), compared with carotid endarterectomy, is emerging as an effective and less invasive method of revascularization for extracranial carotid artery stenosis.

## Case presentation

We report the case of a 67-year-old woman with a 2 year history of recurrent pain in the left upper arm. She was referred to us for further investigation by her GP after noticing a BP difference between arms. Her cardiovascular risk factors include hypertension, type II diabetes mellitus (good glycaemic control with diet) and hyperlipidemia. Her body-mass index was 29kg/m2 and her abdominal circumference was 98cm, consistent with obesity.

From the patient’s history, we could find neither a history of transient loss of consciousness, lightheadedness, transient neurologic deficits, nor chest pain on exertion or at rest.

She was on enalapril (20mg daily), indapamid SR (1.5mg daily), metoprolol (50mg daily), aspirin (75mg daily) and simvastatin (20mg daily).

Her blood pressure was 170/90mmHg at the right arm and 140/80mmHg at the left arm, her heart rate was 64 beats/min, regular, without cardiac murmurs; the physical examination also revealed a low left radial pulse, left supraclavicular, right inguinal and bilateral neck systolic bruits.

The laboratory values on admission included a fasting plasma glucose of 111mg/dL, a total cholesterol of 179mg/dL, with LDL 132mg/dL and HDL 53mg/dL, and a glycated hemoglobin of 5.3%, being otherwise unremarkable.

The electrocardiogram on admission showed sinus rhythm at a rate of 60 beats per minute with nonspecific, flat T waves in V4-6. Chest radiograph showed clear lungs and a normal cardiomediastinal silhouette. A cardiac ultrasonographic examination showed mild, degenerative mitral regurgitation, normal left ventricular function, no enlarged cardiac chambers and no segmental wall-motion abnormalities.

Noninvasive testing of the carotid arteries by duplex ultrasound revealed a severe (>90%) stenosis of the proximal left internal carotid artery and a Doppler signal at the level of the left middle subclavian, axilar and vertebral arteries suggesting a severe distal subclavian artery stenosis with grade 5 vertebral steal. Examination of the right carotid axis was more difficult because of the presence of numerous calcified plaques, but raised suspicion of a severe stenosis at the origin of the right internal carotid artery.

Bilateral selective carotid and subclavian arteriograms showed a 70% stenosis of the right internal carotid artery at its origin, a 30% plaque in the right subclavian artery, a 80-90% stenosis of the left internal carotid artery at its origin and a 80% stenosis in the proximal left subclavian artery. 

Considering the proven atherosclerotic involvement at multiple sites in a patient with several cardiovascular risk factors and at least two-territory atherosclerosis, we found suited to investigate also the coronary arteries. The coronary artery angiogram revealed two serial stenosis of 60-70% in the mid-segment of the left anterior descending artery.

Bilateral staged carotid artery stenting was performed, first left carotid artery followed at 48 hours by right carotid artery plus left subclavian artery, using an embolic protection device approach (FilterWire EZ guide) and self – expanding stents (Carotid Wallstent 9/40mm on the right carotid artery and Protege RX Tapered 7-10/40 mm on the left carotid artery) with pre- and post dilatation, as well as direct left subclavian artery stenting (Dynamic 8/25mm).

The overall final result of the procedure was very good, without major incidents. In the first day after the second procedure the patient’s BP registered a symptomatic fall to 90 mmHg (dizziness) corrected with intravenous saline, which led to temporary discontinuation of the BP lowering agents. 

The patient was discharged on the third day after the second procedure in good health with recommendations for lifestyle changes (low calories, low salt, low fat diet, daily exercise regimen) and medical therapy consisting in dual antiplatelet therapy (aspirin 75mg daily and clopidogrel 75mg daily for three months, then aspirin only), high dose of statin (atorvastatin 40mg daily – with a target for LDL cholesterol < 70mg/dL), ACEi (enalapril 10mg daily with dose titration in order to achieve a BP target of less than 130/80mmHg) and beta-blocker (metoprolol 75 mg daily).

## Discussion

We present the case of a patient with multiple cardiovascular risk factors and atherosclerotic involvement in several important territories, quite paucisymptomatic. Her only complaint was the continuous, non-aggravating and bearable pain in the left upper arm. This case reflects the importance of a thorough clinical examination and complete non-invasive diagnostic work-up, followed by invasive evaluation in a patient with multiple cardiovascular risk factors.

The proximal left subclavian artery is the most commonly diseased arch vessel with significant stenosis (as determined by a systolic blood pressure difference of >15 mmHg between the right and left arm), noted in 7.1% of patients referred to a noninvasive vascular laboratory for any indication. The presence of symptomatic left subclavian artery stenosis is an indication for revascularization. The subclavian artery is a large-diameter vessel with a high flow rate, which makes both stent thrombosis and restenosis rates low (long-term patency 98% at 3 years) [**7**].

The association of asymptomatic bilateral severe internal carotid stenosis is also an indication for revascularization, and choosing between CEA and CAS is mainly based on assessing the risk for surgery (33). As mentioned above, CEA is the current standard of care to prevent stroke in asymptomatic patients with moderate to severe carotid artery stenosis [**8**].

The American Heart Association expert consensus committee recommended that in order to achieve clinical benefit for an asymptomatic patient, from a revascularization procedure (CEA or CAS), the periprocedural threshold for stroke and death should be ≤3% in patients expected to live ≥ 5 years [**9**,**10**].

Carotid artery revascularization in asymptomatic patients has been investigated in single-center [**11**] and multicenter registries [**12**-**17**], nonrandomized comparative trials [**18**, **19**] completed randomized trials [**20**-**26**] and several ongoing randomized trials [**27**, **28**]. In summary, CEA in asymptomatic patients with hemodynamically significant stenoses (60% to 99%) reduces ipsilateral stroke if performed with an acceptable (≤ 3%) perioperative risk of stroke and death, but does not increase the 5-year survival rate. The benefit of CEA in asymptomatic women is not as great as for men.

CAS is an emerging alternative revascularization strategy to prevent stroke. CAS placement is a technique in evolution that includes the recent adoption of distal embolic protection devices and low-profile self-expanding stents. There are specific patient related (age ≥ 75/80 years, dementia, prior/remote stroke, multiple lacunar strokes, renal failure) and lesion related (two or more 90º bends within 5cm of lesion, circumferential calcification ≥ 3 mm in width, intracranial microangiopathy, intravascular filling defect – thrombus, no vascular access) features that increase the risk of stent complications [**10**].

The use of percutaneous techniques to treat coronary diseases has increased over the past two decades. The periprocedural complications of disabling stroke and death with CAS when performed in asymptomatic patients appear to be within or very near the 3% figure established as a surgical risk cutoff.

CAS is an option to be considered in asymptomatic patients with severe (≥ 80%) carotid artery stenosis who are at increased risk of surgical complications (unfavorable anatomic characteristics or medical comorbidities) [**28**]. Currently, there are four major randomized clinical trials comparing carotid artery stenting to CEA [**24**-**27**], with a wide range of operator experience and varying use of distal protection devices, most included patients being symptomatic.

In patients considered of high risk for complications with CEA, the optimal treatment strategy had been investigated in the multicenter SAPPHIRE (Stenting and Angioplasty in Patients at High Risk for Endarterectomy) trial, in which the investigators randomized 334 high-risk patients to CEA versus carotid stenting with distal embolic protection [**24**]. Patients enrolled in this trial were symptomatic with a >50% stenosis or asymptomatic with a >80% stenosis in the internal carotid artery, elderly, and had a high percentage of coronary artery disease (81%). The trial demonstrated non-inferiority of carotid stenting with distal embolic protection when compared to CEA in the primary outcomes of death, ipsilateral stroke, or myocardial infarction at 30 days and ipsilateral stroke and death between 31 days and 1 year.

Based on the SAPPHIRE trial criteria, patients at high risk for CEA are defined as: patients with clinically significant cardiac disease (congestive heart failure, abnormal stress test, or need for open-heart surgery), severe pulmonary disease, contra lateral carotid occlusion, contra lateral laryngeal-nerve palsy, previous radical neck surgery or radiation therapy to the neck, recurrent stenosis after endarterectomy and age >80 years [**24**].

Despite all this trial-derived data, there is still a large category of asymptomatic patients with severe carotid stenosis and average surgical risk, in which we can include our patient, for whom very little information exists. At the present time, there is expert consensus that more data are required, to accept the hypothesis that CAS is non-inferior to CEA in the average-surgical-risk population. Hopefully the two ongoing randomized trials - ACT-1 (Asymptomatic Carotid Trial) and CREST [**30**] - with the purpose of comparing CAS to the traditional and accepted surgical approach CEA for the treatment of carotid artery stenosis to prevent asymptomatic patients - will bring more light on this subject.

Staged percutaneous intervention is the technique of choice in patients with bilateral carotid artery stenosis. The time interval considered appropriate between interventions is of at least 24 hours. Some authors do not like this staged approach, but simultaneous bilateral carotid angioplasty is associated with increased risk for intracerebral hemodynamic related events and symptomatic cerebral edema.

Regarding the existence of asymptomatic one vessel CAD without involvement of the proximal LAD artery and without impairment of left ventricular function at rest, after performing an exercise test which did not show any ST-T changes and was not associated with ischemic symptoms, our attitude was to maximize anti-ischemic medical therapy. The performance of a non-invasive risk stratification investigation is important as high risk results would make coronary revascularization appropriate, low risk findings make it inappropriate, while medium risk patients associate uncertain benefit with coronary revascularization [**32**]. Moreover, it is of high importance that the use of the left internal mammary artery (LIMA) during bypass surgery in patients with severe left subclavian stenosis is associated with coronary-subclavian steal syndrome, leading to reversal of flow in the LIMA graft to the LAD with left arm exertion. This is why, even after percutaneous revascularization of the left subclavian artery, an arterial graft bypass revascularization of the LAD would remain inappropriate. 

## Summary

The presence of multivascular atherosclerotic disease in a diabetic patient with coexisting risk factors is not surprising and it only reinforces the well known fact that we have to search for the involvement of other territories in an oligosymptomatic patient.

Important questions about carotid artery revascularization strategies to prevent stroke still need to be answered. Assessment of the stroke-reduction benefit of optimal modern medical therapy (including atherosclerotic risk factor modification and lifestyle modification) compared with any revascularization strategy for stroke prevention is critical to selecting any treatment strategy.

As proven by existing trials, the comparison of CEA and CAS is a complex issue, Due to variability in patient subsets, differences in end point definitions, changing standards of medical therapy, equipment use (including embolic protection devices and stent use) and differences in operators expertise. 

While acknowledging that much more evidence needs to be gathered, physicians must make decisions and recommendations based on the current available evidence and assessment of the risks and benefits faced by individual patients [**30**].

**Fig. 1:**
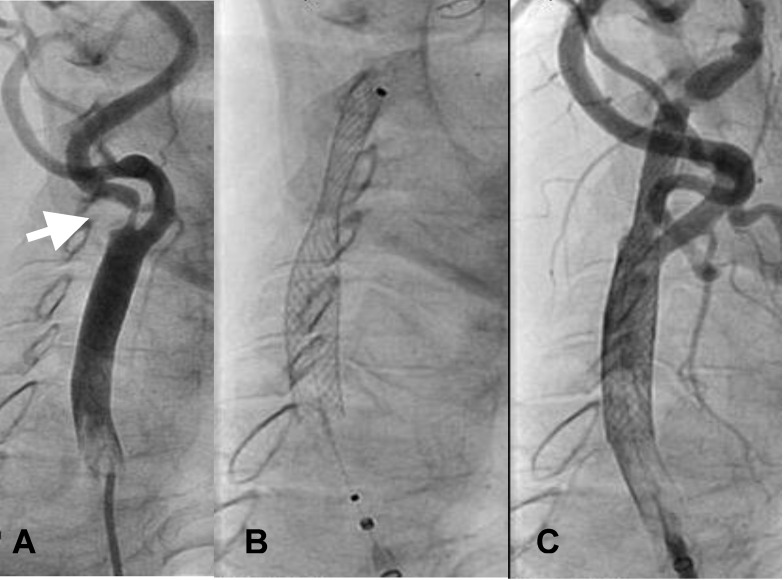
Left carotid arteriogram. A: severe stenosis of the internal carotid artery (arrow). B: auto expandable stent placement. C: injection after stent placement.

**Fig. 2:**
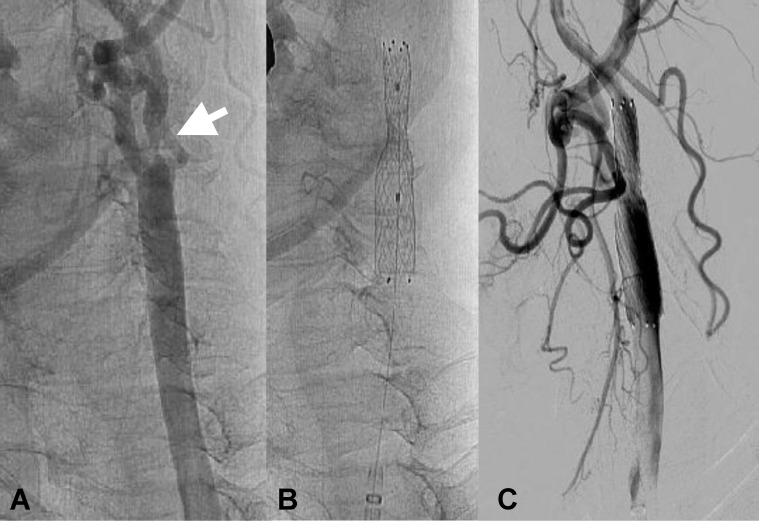
Right carotid arteriogram. A: severe stenosis of the internal carotid artery (arrow). B: auto expandable stent placement. C: injection after stent placement

**Fig. 3:**
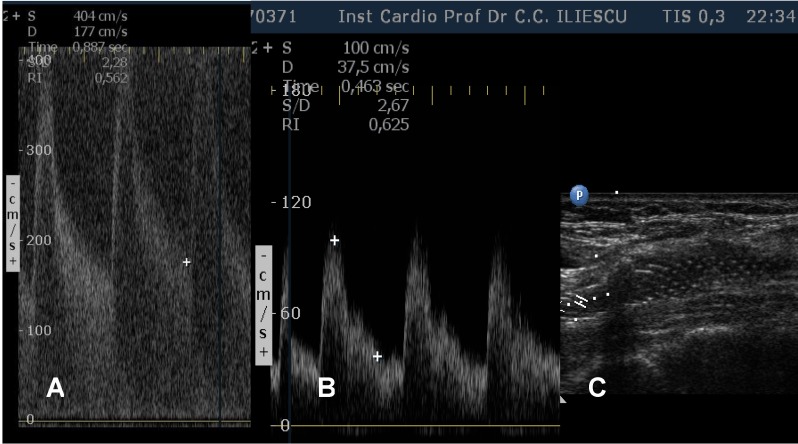
Duplex sonography of the right internal carotid artery. A: high systolic and diastolic intrastenotic velocities suggesting severe stenosis. B: velocities returning to normal after carotid stenting. C: 2D stent visualization.

**Fig. 4:**
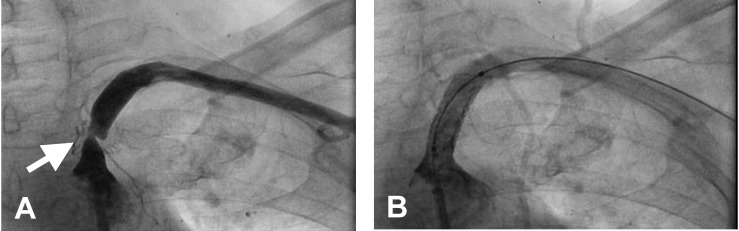
Left subclavian arteriogram. A: severe proximal stenosis (arrow). B: after stent placement (stent and guide wire visible in the lumen).

**Fig. 5:**
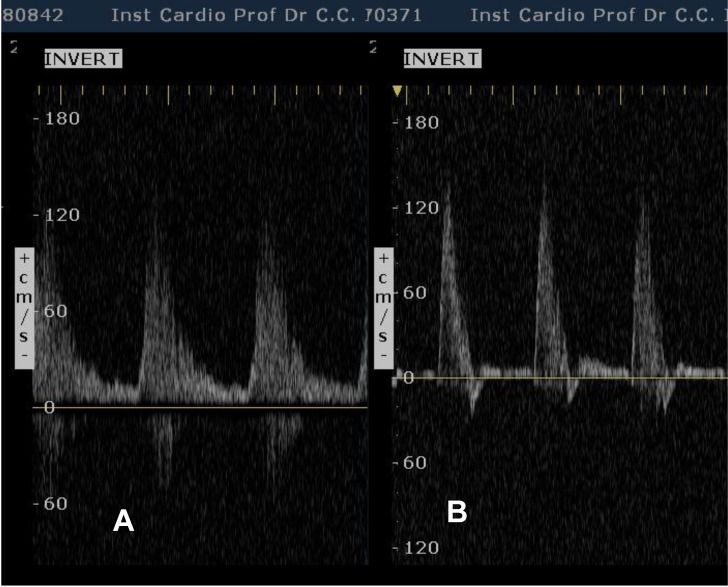
Duplex sonography of the left middle subclavian artery. A: monophasic waveform compatible with poststenotic flow. B: normal triphasic waveform after angioplasty.

**Fig. 6:**
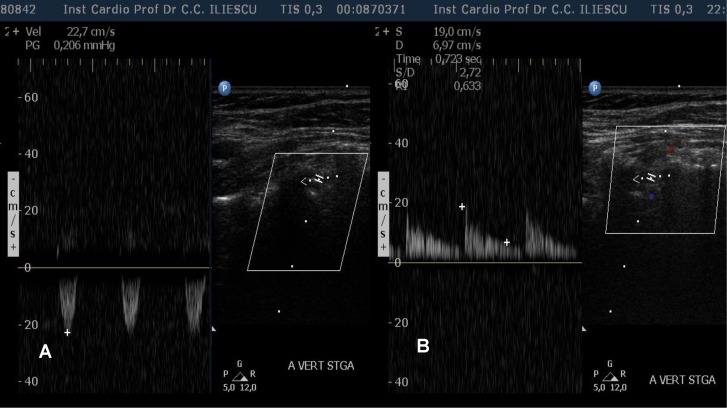
Duplex sonography of the left vertebral artery. A: systolic reversal of flow (grade 5 vertebral steal) without diastolic velocities. B: normal biphasic Doppler waveforms after left subclavian artery stent placement.
